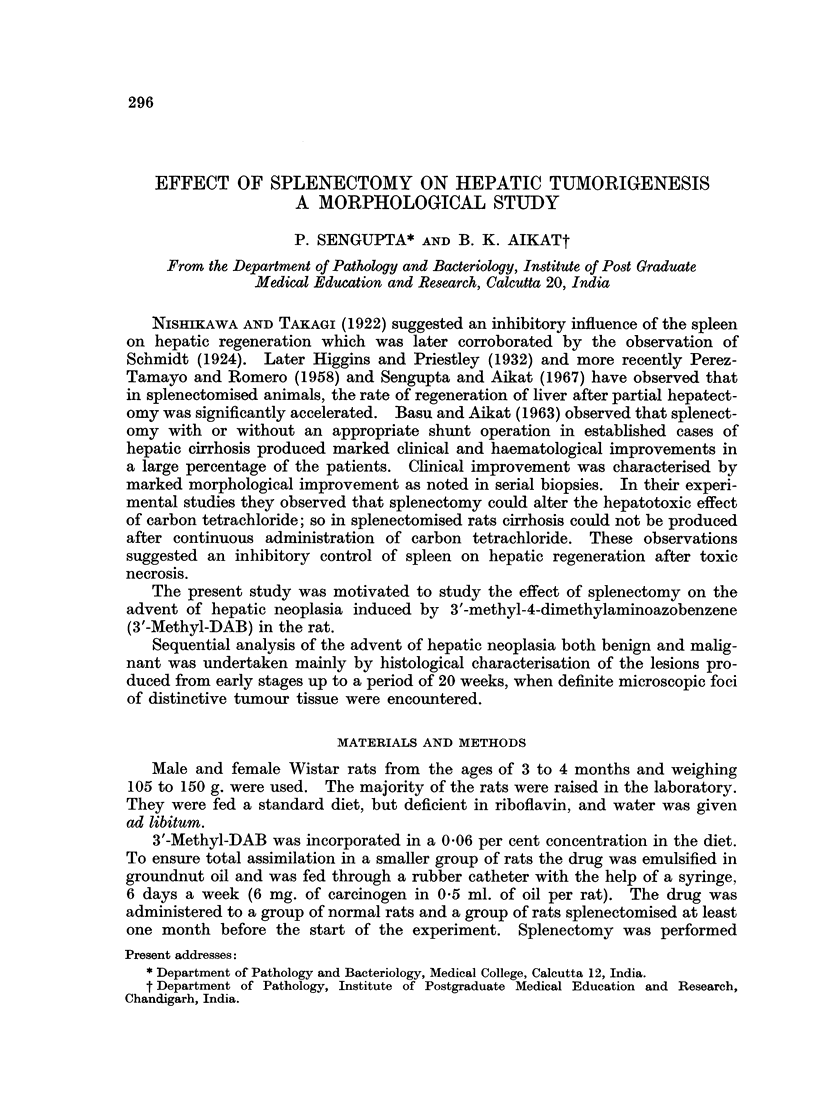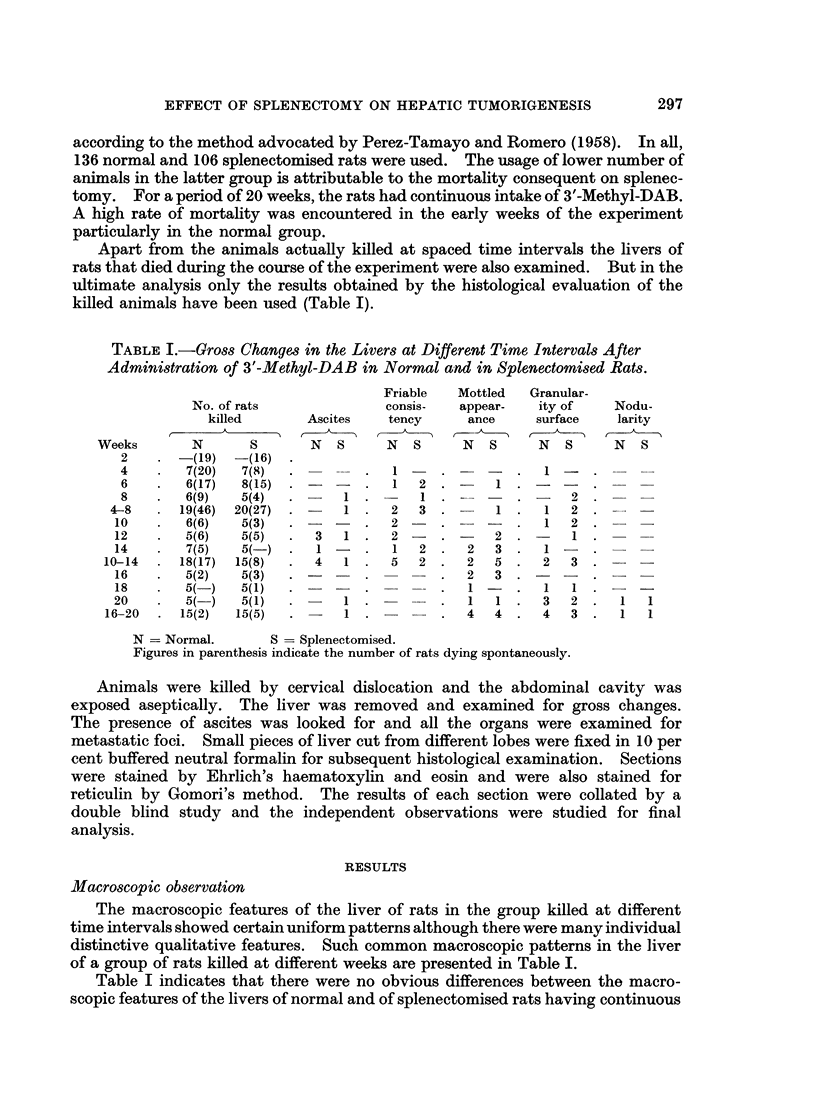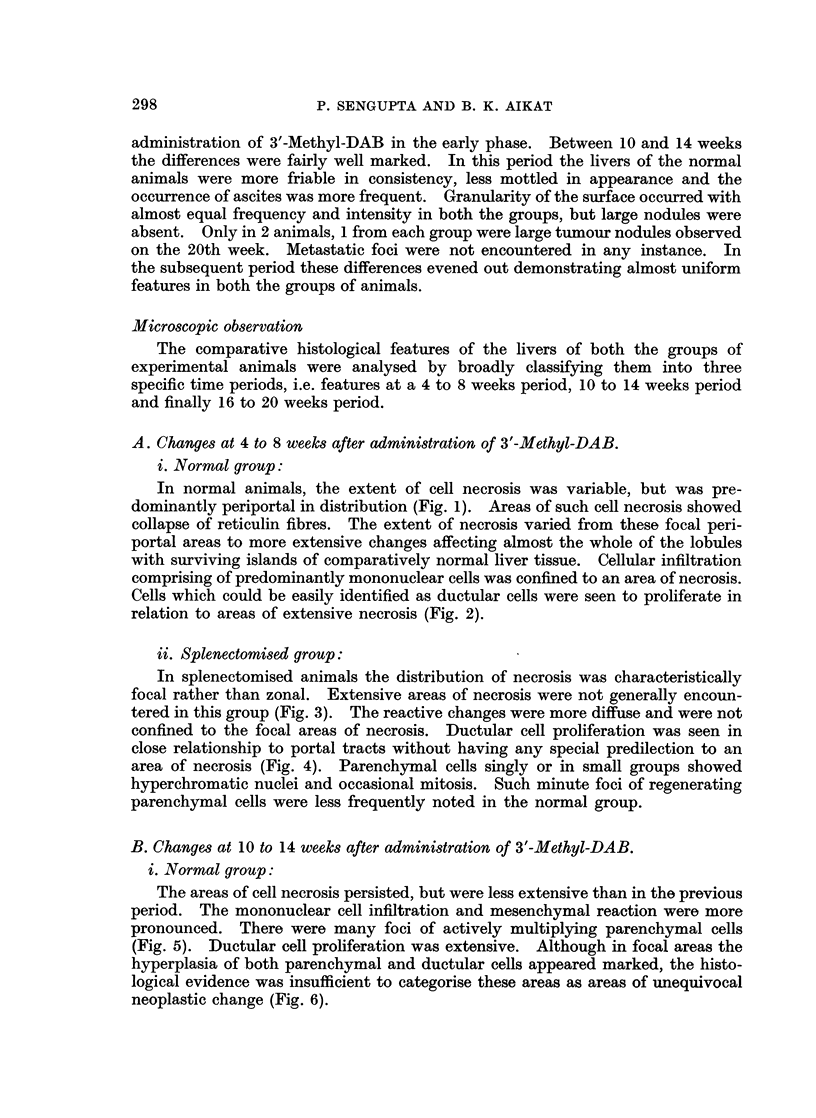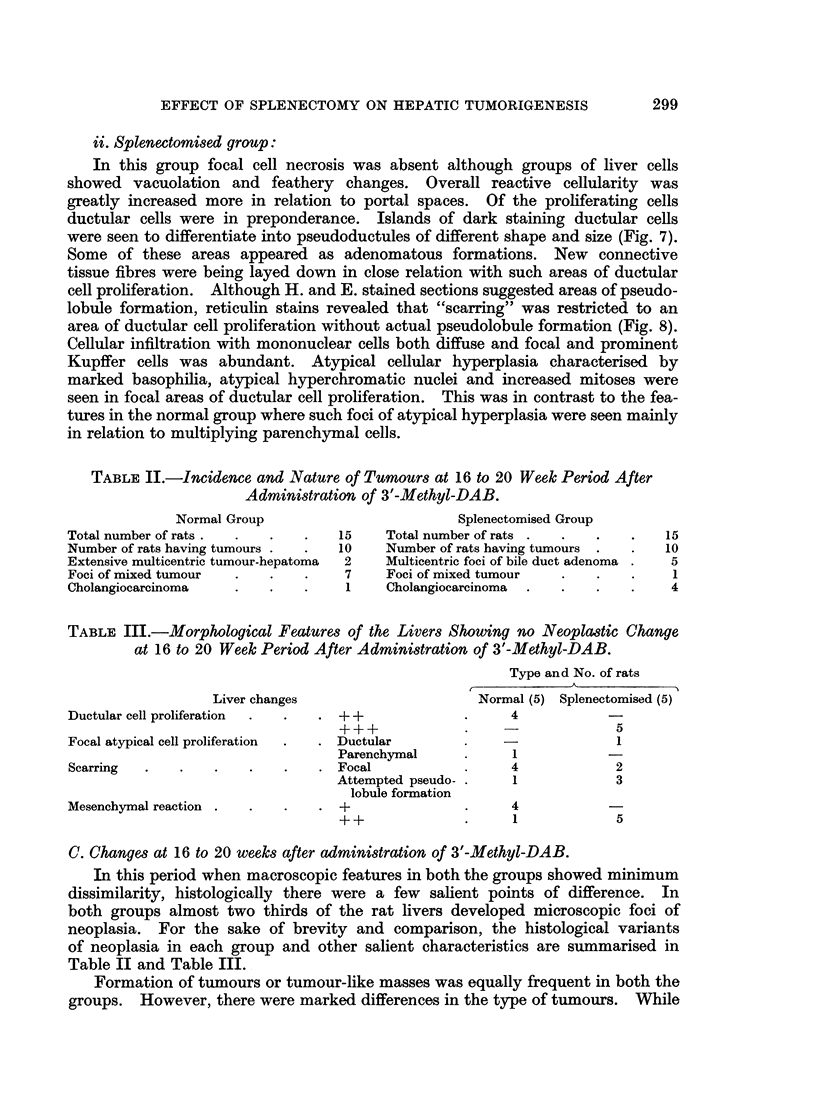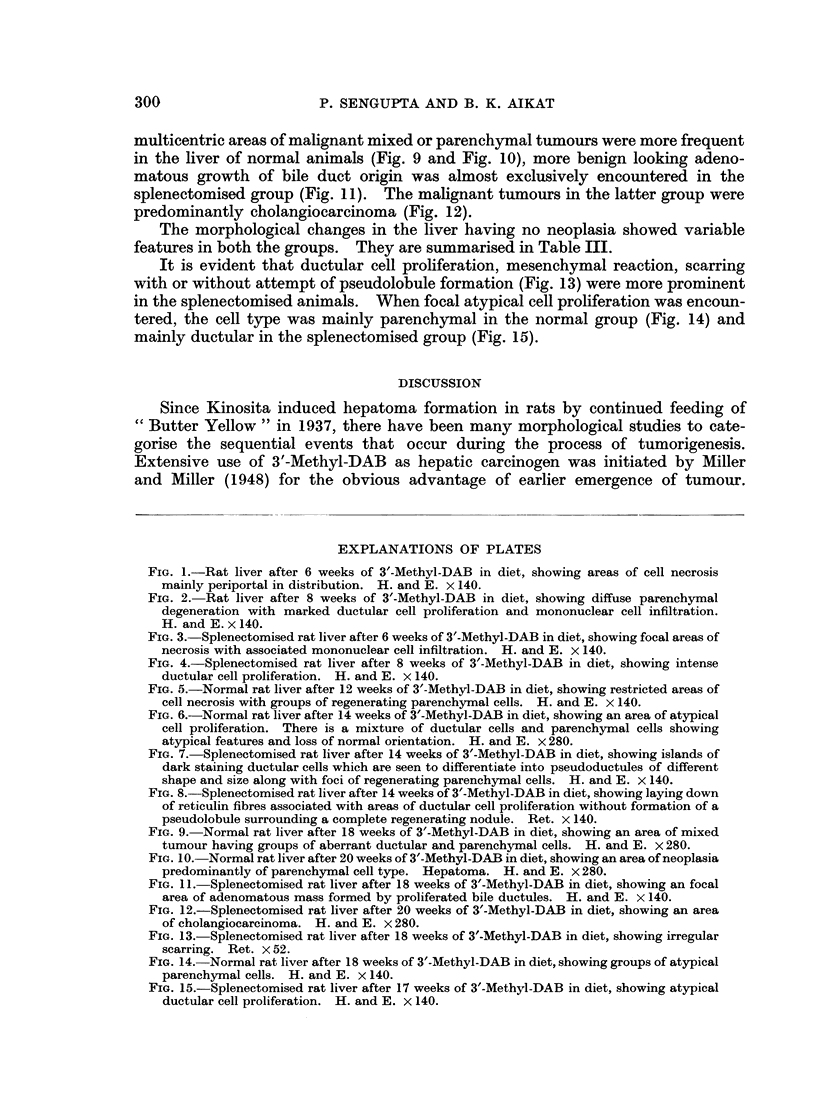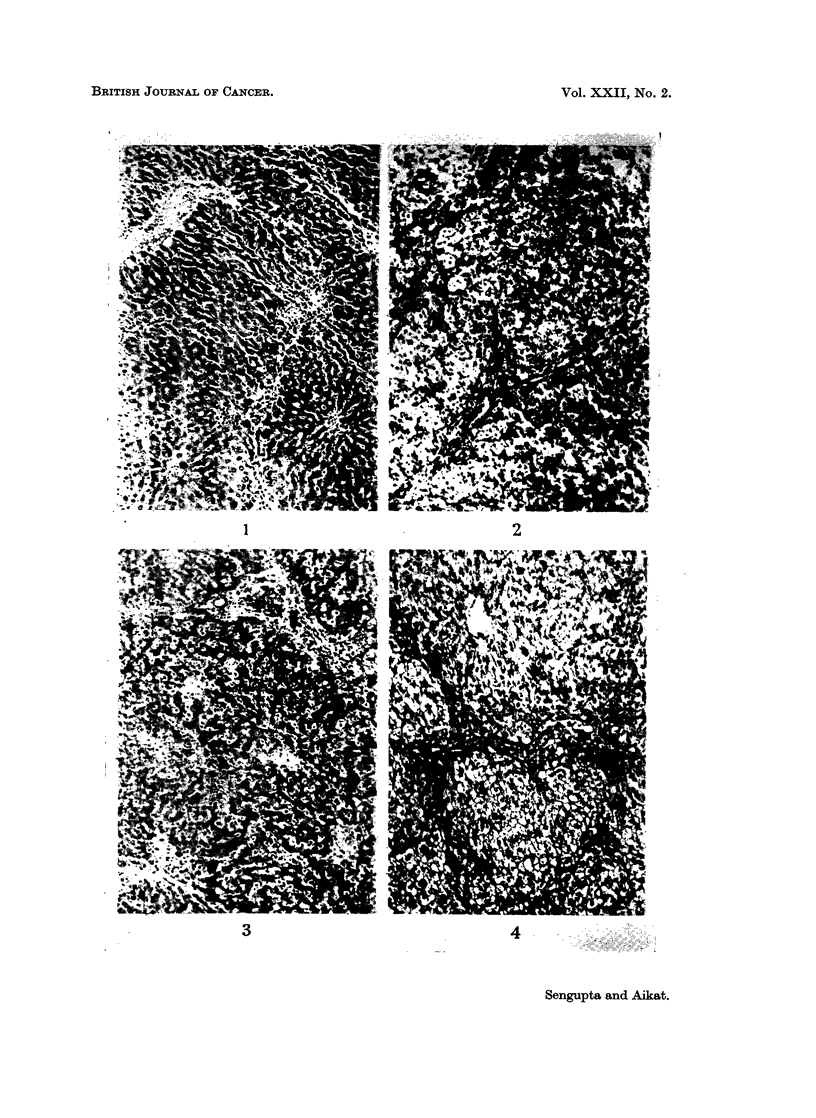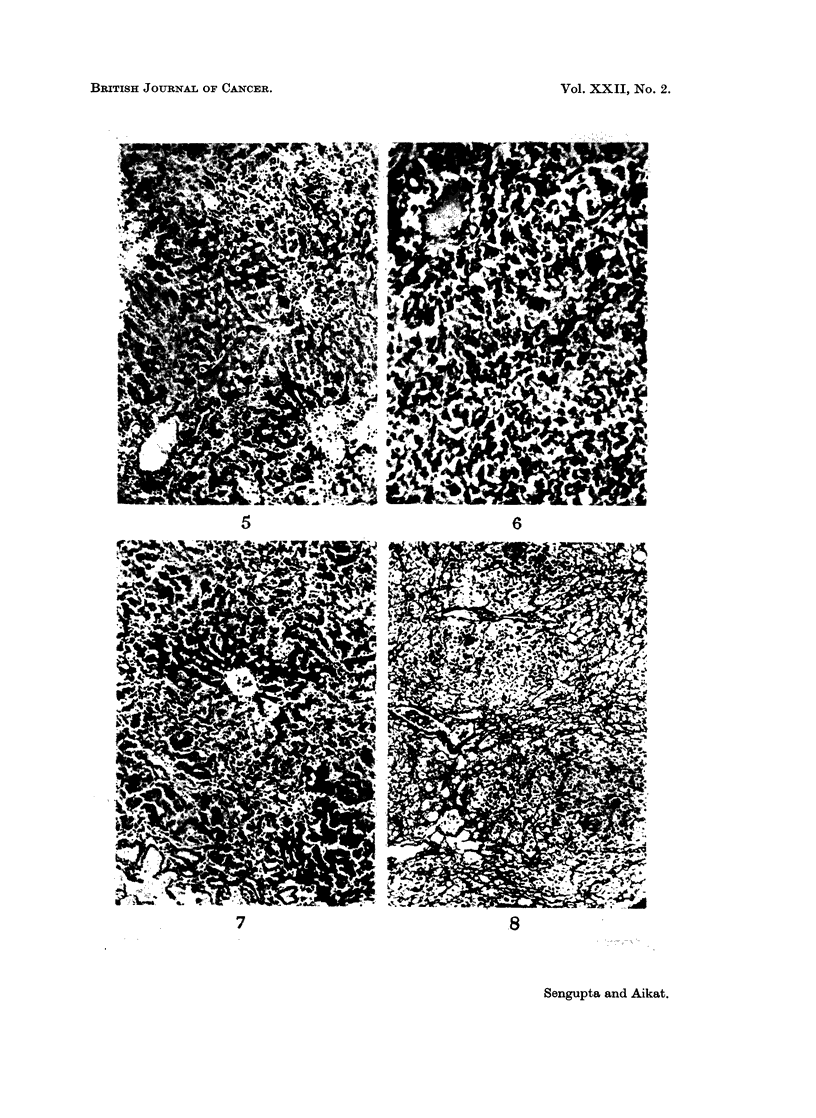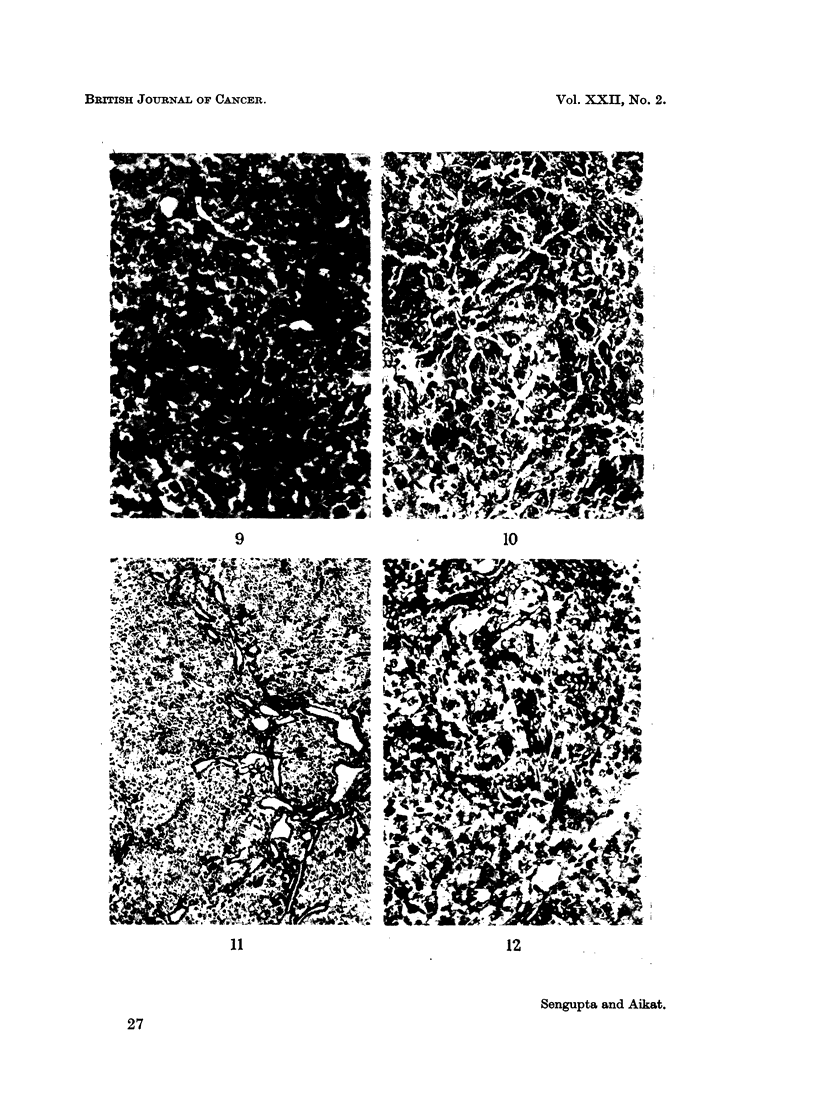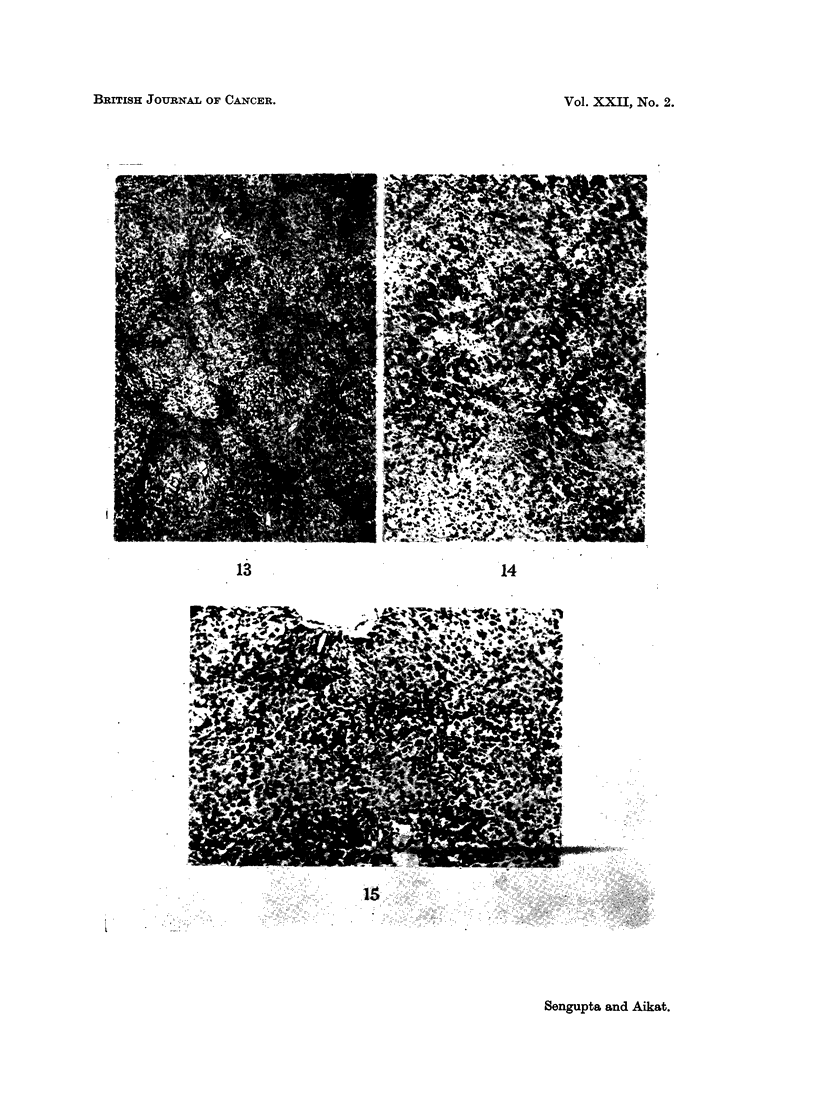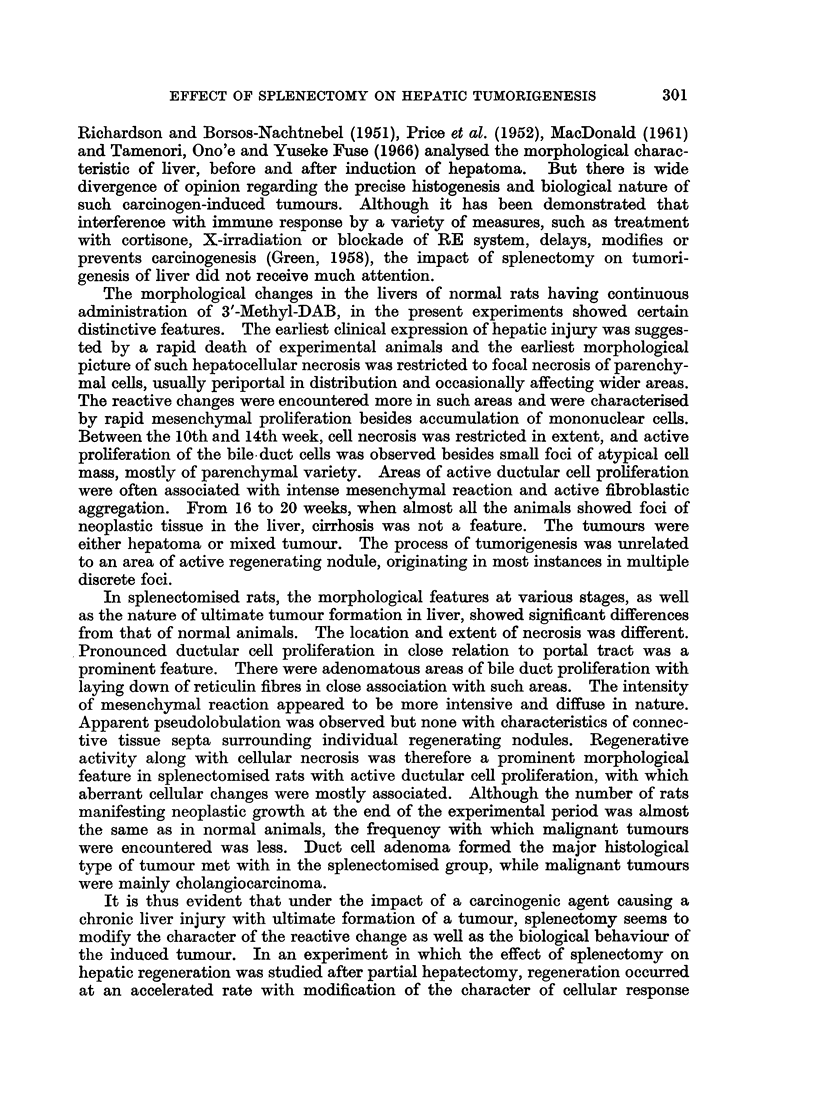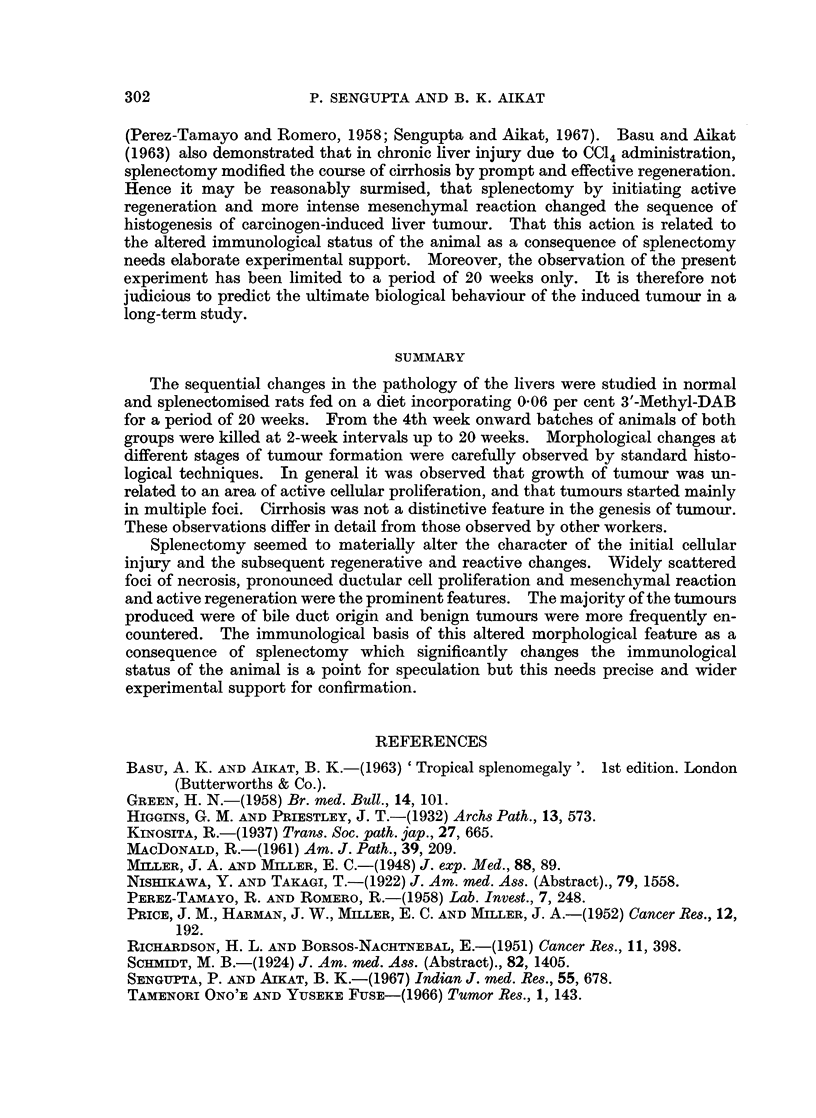# Effect of splenectomy on hepatic tumorigenesis. A morphological study.

**DOI:** 10.1038/bjc.1968.37

**Published:** 1968-06

**Authors:** P. Sengupta, B. K. Aikat

## Abstract

**Images:**


### EFFECT OF SPLENECTOMY ON HEPATIC TUMORIGENESIS A MORPHOLOGICAL STUDY

P. SENGUPTA* AND B. K. AIKAT†

*From the Department of Pathology and Bacteriology, Institute of Post Graduate Medical Education and Research, Calcutta 20, India*

NISHIKAWA AND TAKAGI (1922) suggested an inhibitory influence of the spleen on hepatic regeneration which was later corroborated by the observation of Schmidt (1924). Later Higgins and Priestley (1932) and more recently Perez-Tamayo and Romero (1958) and Sengupta and Aikat (1967) have observed that in splenectomised animals, the rate of regeneration of liver after partial hepatectomy was significantly accelerated. Basu and Aikat (1963) observed that splenectomy with or without an appropriate shunt operation in established cases of hepatic cirrhosis produced marked clinical and haematological improvements in a large percentage of the patients. Clinical improvement was characterised by marked morphological improvement as noted in serial biopsies. In their experimental studies they observed that splenectomy could alter the hepatotoxic effect of carbon tetrachloride; so in splenectomised rats cirrhosis could not be produced after continuous administration of carbon tetrachloride. These observations suggested an inhibitory control of spleen on hepatic regeneration after toxic necrosis.

The present study was motivated to study the effect of splenectomy on the advent of hepatic neoplasia induced by 3′-methyl-4-dimethylaminoazobenzene (3′-Methyl-DAB) in the rat.

Sequential analysis of the advent of hepatic neoplasia both benign and malignant was undertaken mainly by histological characterisation of the lesions produced from early stages up to a period of 20 weeks, when definite microscopic foci of distinctive tumour tissue were encountered.

### MATERIALS AND METHODS

Male and female Wistar rats from the ages of 3 to 4 months and weighing 105 to 150 g. were used. The majority of the rats were raised in the laboratory. They were fed a standard diet, but deficient in riboflavin, and water was given *ad libitum*.

3′-Methyl-DAB was incorporated in a 0·06 per cent concentration in the diet. To ensure total assimilation in a smaller group of rats the drug was emulsified in groundnut oil and was fed through a rubber catheter with the help of a syringe, 6 days a week (6 mg. of carcinogen in 0·5 ml. of oil per rat). The drug was administered to a group of normal rats and a group of rats splenectomised at least one month before the start of the experiment. Splenectomy was performed

Present addresses:

* Department of Pathology and Bacteriology, Medical College, Calcutta 12, India.

† Department of Pathology, Institute of Postgraduate Medical Education and Research, Chandigarh, India.

according to the method advocated by Perez-Tamayo and Romero (1958). In all, 136 normal and 106 splenectomised rats were used. The usage of lower number of animals in the latter group is attributable to the mortality consequent on splenectomy. For a period of 20 weeks, the rats had continuous intake of 3′-Methyl-DAB. A high rate of mortality was encountered in the early weeks of the experiment particularly in the normal group.

Apart from the animals actually killed at spaced time intervals the livers of rats that died during the course of the experiment were also examined. But in the ultimate analysis only the results obtained by the histological evaluation of the killed animals have been used (Table I).

TABLE I.—*Gross Changes in the Livers at Different Time Intervals After Administration of 3′-Methyl-DAB in Normal and in Splenectomised Rats.*

| | No. of rats killed | | Ascites | | Friable consistency | | Mottled appearance | | Granularity of surface | | Nodularity | |
|---|---|---|---|---|---|---|---|---|---|---|---|---|
| Weeks | N | S | N | S | N | S | N | S | N | S | N | S |
| 2 | —(19) | —(16) | | | | | | | | | | |
| 4 | 7(20) | 7(8) | — | — | 1 | — | — | — | 1 | — | — | — |
| 6 | 6(17) | 8(15) | — | — | 1 | 2 | — | 1 | — | — | — | — |
| 8 | 6(9) | 5(4) | — | 1 | — | 1 | — | — | — | 2 | — | — |
| 4–8 | 19(46) | 20(27) | — | 1 | 2 | 3 | — | 1 | 1 | 2 | — | — |
| 10 | 6(6) | 5(3) | — | — | 2 | — | — | — | 1 | 2 | — | — |
| 12 | 5(6) | 5(5) | 3 | 1 | 2 | — | — | 2 | — | 1 | — | — |
| 14 | 7(5) | 5(—) | 1 | — | 1 | 2 | 2 | 3 | 1 | — | — | — |
| 10–14 | 18(17) | 15(8) | 4 | 1 | 5 | 2 | 2 | 5 | 2 | 3 | — | — |
| 16 | 5(2) | 5(3) | — | — | — | — | 2 | 3 | — | — | — | — |
| 18 | 5(—) | 5(1) | — | — | — | — | 1 | — | 1 | 1 | — | — |
| 20 | 5(—) | 5(1) | — | 1 | — | — | 1 | 1 | 3 | 2 | 1 | 1 |
| 16–20 | 15(2) | 15(5) | — | 1 | — | — | 4 | 4 | 4 | 3 | 1 | 1 |

N = Normal. S = Splenectomised.
Figures in parenthesis indicate the number of rats dying spontaneously.

Animals were killed by cervical dislocation and the abdominal cavity was exposed aseptically. The liver was removed and examined for gross changes. The presence of ascites was looked for and all the organs were examined for metastatic foci. Small pieces of liver cut from different lobes were fixed in 10 per cent buffered neutral formalin for subsequent histological examination. Sections were stained by Ehrlich's haematoxylin and eosin and were also stained for reticulin by Gomori's method. The results of each section were collated by a double blind study and the independent observations were studied for final analysis.

### RESULTS

### *Macroscopic observation*

The macroscopic features of the liver of rats in the group killed at different time intervals showed certain uniform patterns although there were many individual distinctive qualitative features. Such common macroscopic patterns in the liver of a group of rats killed at different weeks are presented in Table I.

Table I indicates that there were no obvious differences between the macroscopic features of the livers of normal and of splenectomised rats having continuous administration of 3′-Methyl-DAB in the early phase. Between 10 and 14 weeks the differences were fairly well marked. In this period the livers of the normal animals were more friable in consistency, less mottled in appearance and the occurrence of ascites was more frequent. Granularity of the surface occurred with almost equal frequency and intensity in both the groups, but large nodules were absent. Only in 2 animals, 1 from each group were large tumour nodules observed on the 20th week. Metastatic foci were not encountered in any instance. In the subsequent period these differences evened out demonstrating almost uniform features in both the groups of animals.

*Microscopic observation*

The comparative histological features of the livers of both the groups of experimental animals were analysed by broadly classifying them into three specific time periods, i.e. features at a 4 to 8 weeks period, 10 to 14 weeks period and finally 16 to 20 weeks period.

*A. Changes at* 4 *to* 8 *weeks after administration of* 3′-*Methyl-DAB.*

*i. Normal group:*

In normal animals, the extent of cell necrosis was variable, but was predominantly periportal in distribution (Fig. 1). Areas of such cell necrosis showed collapse of reticulin fibres. The extent of necrosis varied from these focal periportal areas to more extensive changes affecting almost the whole of the lobules with surviving islands of comparatively normal liver tissue. Cellular infiltration comprising of predominantly mononuclear cells was confined to an area of necrosis. Cells which could be easily identified as ductular cells were seen to proliferate in relation to areas of extensive necrosis (Fig. 2).

*ii. Splenectomised group:*

In splenectomised animals the distribution of necrosis was characteristically focal rather than zonal. Extensive areas of necrosis were not generally encountered in this group (Fig. 3). The reactive changes were more diffuse and were not confined to the focal areas of necrosis. Ductular cell proliferation was seen in close relationship to portal tracts without having any special predilection to an area of necrosis (Fig. 4). Parenchymal cells singly or in small groups showed hyperchromatic nuclei and occasional mitosis. Such minute foci of regenerating parenchymal cells were less frequently noted in the normal group.

*B. Changes at* 10 *to* 14 *weeks after administration of* 3′-*Methyl-DAB.*

*i. Normal group:*

The areas of cell necrosis persisted, but were less extensive than in the previous period. The mononuclear cell infiltration and mesenchymal reaction were more pronounced. There were many foci of actively multiplying parenchymal cells (Fig. 5). Ductular cell proliferation was extensive. Although in focal areas the hyperplasia of both parenchymal and ductular cells appeared marked, the histological evidence was insufficient to categorise these areas as areas of unequivocal neoplastic change (Fig. 6).

*ii. Splenectomised group:*

In this group focal cell necrosis was absent although groups of liver cells showed vacuolation and feathery changes. Overall reactive cellularity was greatly increased more in relation to portal spaces. Of the proliferating cells ductular cells were in preponderance. Islands of dark staining ductular cells were seen to differentiate into pseudoductules of different shape and size (Fig. 7). Some of these areas appeared as adenomatous formations. New connective tissue fibres were being layed down in close relation with such areas of ductular cell proliferation. Although H. and E. stained sections suggested areas of pseudolobule formation, reticulin stains revealed that "scarring" was restricted to an area of ductular cell proliferation without actual pseudolobule formation (Fig. 8). Cellular infiltration with mononuclear cells both diffuse and focal and prominent Kupffer cells was abundant. Atypical cellular hyperplasia characterised by marked basophilia, atypical hyperchromatic nuclei and increased mitoses were seen in focal areas of ductular cell proliferation. This was in contrast to the features in the normal group where such foci of atypical hyperplasia were seen mainly in relation to multiplying parenchymal cells.

TABLE II.—*Incidence and Nature of Tumours at* 16 *to* 20 *Week Period After Administration of* 3'-*Methyl-DAB.*

| Normal Group | | Splenectomised Group | |
|---|---|---|---|
| Total number of rats . . . . | 15 | Total number of rats . . . . | 15 |
| Number of rats having tumours . . | 10 | Number of rats having tumours . . | 10 |
| Extensive multicentric tumour-hepatoma | 2 | Multicentric foci of bile duct adenoma . | 5 |
| Foci of mixed tumour . . . | 7 | Foci of mixed tumour . . . | 1 |
| Cholangiocarcinoma . . . | 1 | Cholangiocarcinoma . . . . | 4 |

TABLE III.—*Morphological Features of the Livers Showing no Neoplastic Change at* 16 *to* 20 *Week Period After Administration of* 3'-*Methyl-DAB.*

| | | Type and No. of rats | |
|---|---|---|---|
| Liver changes | | Normal (5) | Splenectomised (5) |
| Ductular cell proliferation . . . | ++ | 4 | — |
| | +++ | — | 5 |
| Focal atypical cell proliferation . . | Ductular | — | 1 |
| | Parenchymal | 1 | — |
| Scarring . . . . . . | Focal | 4 | 2 |
| | Attempted pseudo-lobule formation | 1 | 3 |
| Mesenchymal reaction . . . . | + | 4 | — |
| | ++ | 1 | 5 |

*C. Changes at* 16 *to* 20 *weeks after administration of* 3'-*Methyl-DAB.*

In this period when macroscopic features in both the groups showed minimum dissimilarity, histologically there were a few salient points of difference. In both groups almost two thirds of the rat livers developed microscopic foci of neoplasia. For the sake of brevity and comparison, the histological variants of neoplasia in each group and other salient characteristics are summarised in Table II and Table III.

Formation of tumours or tumour-like masses was equally frequent in both the groups. However, there were marked differences in the type of tumours. While multicentric areas of malignant mixed or parenchymal tumours were more frequent in the liver of normal animals (Fig. 9 and Fig. 10), more benign looking adenomatous growth of bile duct origin was almost exclusively encountered in the splenectomised group (Fig. 11). The malignant tumours in the latter group were predominantly cholangiocarcinoma (Fig. 12).

The morphological changes in the liver having no neoplasia showed variable features in both the groups. They are summarised in Table III.

It is evident that ductular cell proliferation, mesenchymal reaction, scarring with or without attempt of pseudolobule formation (Fig. 13) were more prominent in the splenectomised animals. When focal atypical cell proliferation was encountered, the cell type was mainly parenchymal in the normal group (Fig. 14) and mainly ductular in the splenectomised group (Fig. 15).

### DISCUSSION

Since Kinosita induced hepatoma formation in rats by continued feeding of " Butter Yellow " in 1937, there have been many morphological studies to categorise the sequential events that occur during the process of tumorigenesis. Extensive use of 3′-Methyl-DAB as hepatic carcinogen was initiated by Miller and Miller (1948) for the obvious advantage of earlier emergence of tumour.

---

### EXPLANATIONS OF PLATES

FIG. 1.—Rat liver after 6 weeks of 3′-Methyl-DAB in diet, showing areas of cell necrosis mainly periportal in distribution. H. and E. ×140.

FIG. 2.—Rat liver after 8 weeks of 3′-Methyl-DAB in diet, showing diffuse parenchymal degeneration with marked ductular cell proliferation and mononuclear cell infiltration. H. and E. ×140.

FIG. 3.—Splenectomised rat liver after 6 weeks of 3′-Methyl-DAB in diet, showing focal areas of necrosis with associated mononuclear cell infiltration. H. and E. ×140.

FIG. 4.—Splenectomised rat liver after 8 weeks of 3′-Methyl-DAB in diet, showing intense ductular cell proliferation. H. and E. ×140.

FIG. 5.—Normal rat liver after 12 weeks of 3′-Methyl-DAB in diet, showing restricted areas of cell necrosis with groups of regenerating parenchymal cells. H. and E. ×140.

FIG. 6.—Normal rat liver after 14 weeks of 3′-Methyl-DAB in diet, showing an area of atypical cell proliferation. There is a mixture of ductular cells and parenchymal cells showing atypical features and loss of normal orientation. H. and E. ×280.

FIG. 7.—Splenectomised rat liver after 14 weeks of 3′-Methyl-DAB in diet, showing islands of dark staining ductular cells which are seen to differentiate into pseudoductules of different shape and size along with foci of regenerating parenchymal cells. H. and E. ×140.

FIG. 8.—Splenectomised rat liver after 14 weeks of 3′-Methyl-DAB in diet, showing laying down of reticulin fibres associated with areas of ductular cell proliferation without formation of a pseudolobule surrounding a complete regenerating nodule. Ret. ×140.

FIG. 9.—Normal rat liver after 18 weeks of 3′-Methyl-DAB in diet, showing an area of mixed tumour having groups of aberrant ductular and parenchymal cells. H. and E. ×280.

FIG. 10.—Normal rat liver after 20 weeks of 3′-Methyl-DAB in diet, showing an area of neoplasia predominantly of parenchymal cell type. Hepatoma. H. and E. ×280.

FIG. 11.—Splenectomised rat liver after 18 weeks of 3′-Methyl-DAB in diet, showing an focal area of adenomatous mass formed by proliferated bile ductules. H. and E. ×140.

FIG. 12.—Splenectomised rat liver after 20 weeks of 3′-Methyl-DAB in diet, showing an area of cholangiocarcinoma. H. and E. ×280.

FIG. 13.—Splenectomised rat liver after 18 weeks of 3′-Methyl-DAB in diet, showing irregular scarring. Ret. ×52.

FIG. 14.—Normal rat liver after 18 weeks of 3′-Methyl-DAB in diet, showing groups of atypical parenchymal cells. H. and E. ×140.

FIG. 15.—Splenectomised rat liver after 17 weeks of 3′-Methyl-DAB in diet, showing atypical ductular cell proliferation. H. and E. ×140.

Sengupta and Aikat.

5

6

7

8

Sengupta and Aikat.

9 10 11 12

Sengupta and Aikat.

13

14

15

Richardson and Borsos-Nachtnebel (1951), Price *et al.* (1952), MacDonald (1961) and Tamenori, Ono'e and Yuseke Fuse (1966) analysed the morphological characteristic of liver, before and after induction of hepatoma. But there is wide divergence of opinion regarding the precise histogenesis and biological nature of such carcinogen-induced tumours. Although it has been demonstrated that interference with immune response by a variety of measures, such as treatment with cortisone, X-irradiation or blockade of RE system, delays, modifies or prevents carcinogenesis (Green, 1958), the impact of splenectomy on tumorigenesis of liver did not receive much attention.

The morphological changes in the livers of normal rats having continuous administration of 3′-Methyl-DAB, in the present experiments showed certain distinctive features. The earliest clinical expression of hepatic injury was suggested by a rapid death of experimental animals and the earliest morphological picture of such hepatocellular necrosis was restricted to focal necrosis of parenchymal cells, usually periportal in distribution and occasionally affecting wider areas. The reactive changes were encountered more in such areas and were characterised by rapid mesenchymal proliferation besides accumulation of mononuclear cells. Between the 10th and 14th week, cell necrosis was restricted in extent, and active proliferation of the bile duct cells was observed besides small foci of atypical cell mass, mostly of parenchymal variety. Areas of active ductular cell proliferation were often associated with intense mesenchymal reaction and active fibroblastic aggregation. From 16 to 20 weeks, when almost all the animals showed foci of neoplastic tissue in the liver, cirrhosis was not a feature. The tumours were either hepatoma or mixed tumour. The process of tumorigenesis was unrelated to an area of active regenerating nodule, originating in most instances in multiple discrete foci.

In splenectomised rats, the morphological features at various stages, as well as the nature of ultimate tumour formation in liver, showed significant differences from that of normal animals. The location and extent of necrosis was different. Pronounced ductular cell proliferation in close relation to portal tract was a prominent feature. There were adenomatous areas of bile duct proliferation with laying down of reticulin fibres in close association with such areas. The intensity of mesenchymal reaction appeared to be more intensive and diffuse in nature. Apparent pseudolobulation was observed but none with characteristics of connective tissue septa surrounding individual regenerating nodules. Regenerative activity along with cellular necrosis was therefore a prominent morphological feature in splenectomised rats with active ductular cell proliferation, with which aberrant cellular changes were mostly associated. Although the number of rats manifesting neoplastic growth at the end of the experimental period was almost the same as in normal animals, the frequency with which malignant tumours were encountered was less. Duct cell adenoma formed the major histological type of tumour met with in the splenectomised group, while malignant tumours were mainly cholangiocarcinoma.

It is thus evident that under the impact of a carcinogenic agent causing a chronic liver injury with ultimate formation of a tumour, splenectomy seems to modify the character of the reactive change as well as the biological behaviour of the induced tumour. In an experiment in which the effect of splenectomy on hepatic regeneration was studied after partial hepatectomy, regeneration occurred at an accelerated rate with modification of the character of cellular response

(Perez-Tamayo and Romero, 1958; Sengupta and Aikat, 1967). Basu and Aikat (1963) also demonstrated that in chronic liver injury due to $CCl_4$ administration, splenectomy modified the course of cirrhosis by prompt and effective regeneration. Hence it may be reasonably surmised, that splenectomy by initiating active regeneration and more intense mesenchymal reaction changed the sequence of histogenesis of carcinogen-induced liver tumour. That this action is related to the altered immunological status of the animal as a consequence of splenectomy needs elaborate experimental support. Moreover, the observation of the present experiment has been limited to a period of 20 weeks only. It is therefore not judicious to predict the ultimate biological behaviour of the induced tumour in a long-term study.

### SUMMARY

The sequential changes in the pathology of the livers were studied in normal and splenectomised rats fed on a diet incorporating 0·06 per cent 3′-Methyl-DAB for a period of 20 weeks. From the 4th week onward batches of animals of both groups were killed at 2-week intervals up to 20 weeks. Morphological changes at different stages of tumour formation were carefully observed by standard histological techniques. In general it was observed that growth of tumour was unrelated to an area of active cellular proliferation, and that tumours started mainly in multiple foci. Cirrhosis was not a distinctive feature in the genesis of tumour. These observations differ in detail from those observed by other workers.

Splenectomy seemed to materially alter the character of the initial cellular injury and the subsequent regenerative and reactive changes. Widely scattered foci of necrosis, pronounced ductular cell proliferation and mesenchymal reaction and active regeneration were the prominent features. The majority of the tumours produced were of bile duct origin and benign tumours were more frequently encountered. The immunological basis of this altered morphological feature as a consequence of splenectomy which significantly changes the immunological status of the animal is a point for speculation but this needs precise and wider experimental support for confirmation.

### REFERENCES

Basu, A. K. and Aikat, B. K.—(1963) ‘ Tropical splenomegaly ’. 1st edition. London (Butterworths & Co.).

Green, H. N.—(1958) *Br. med. Bull.*, **14**, 101.

Higgins, G. M. and Priestley, J. T.—(1932) *Archs Path.*, **13**, 573.

Kinosita, R.—(1937) *Trans. Soc. path. jap.*, **27**, 665.

MacDonald, R.—(1961) *Am. J. Path.*, **39**, 209.

Miller, J. A. and Miller, E. C.—(1948) *J. exp. Med.*, **88**, 89.

Nishikawa, Y. and Takagi, T.—(1922) *J. Am. med. Ass.* (Abstract)., **79**, 1558.

Perez-Tamayo, R. and Romero, R.—(1958) *Lab. Invest.*, **7**, 248.

Price, J. M., Harman, J. W., Miller, E. C. and Miller, J. A.—(1952) *Cancer Res.*, **12**, 192.

Richardson, H. L. and Borsos-Nachtnebal, E.—(1951) *Cancer Res.*, **11**, 398.

Schmidt, M. B.—(1924) *J. Am. med. Ass.* (Abstract)., **82**, 1405.

Sengupta, P. and Aikat, B. K.—(1967) *Indian J. med. Res.*, **55**, 678.

Tamenori Ono’e and Yuseke Fuse—(1966) *Tumor Res.*, **1**, 143.